# Efficacy and safety of turmeric and curcumin in lowering blood lipid levels in patients with cardiovascular risk factors: a meta-analysis of randomized controlled trials

**DOI:** 10.1186/s12937-017-0293-y

**Published:** 2017-10-11

**Authors:** Si Qin, Lifan Huang, Jiaojiao Gong, Shasha Shen, Juan Huang, Hong Ren, Huaidong Hu

**Affiliations:** 1 Department of Clinical Nutrition, The Second Affiliated Hospital of Chongqing Medical University, No.76, Linjiang Road, Chongqing, 400010 People’s Republic of China; 2 Department of Infectious Diseases, Institute for Viral Hepatitis, Key Laboratory of Molecular Biology for Infectious Diseases (Ministry of Education), Institute for Viral Hepatitis, The Second Affiliated Hospital of Chongqing Medical University, Chongqing, People’s Republic of China

**Keywords:** Turmeric, Curcumin, Cholesterol, Cardiovascular risk, Triglycerides, Meta-analysis

## Abstract

**Background:**

Dyslipidemia is an important and common cardiovascular risk factor in the general population. The lipid-lowering effects of turmeric and curcumin are unconfirmed. We performed a meta-analysis to assess the efficacy and safety of turmeric and curcumin in lowering blood lipids in patients at risk of cardiovascular disease (CVD).

**Methods:**

A comprehensive literature search was conducted on PubMed, Embase, Ovid, Medline and Cochrane Library databases to identify randomized controlled trials (published as of November 2016) that assessed the effect of turmeric and curcumin on blood lipid levels including total cholesterol (TC), low-density lipoprotein cholesterol (LDL-C), high-density lipoprotein cholesterol (HDL-C), and triglycerides (TG). Pooled standardized mean difference (SMD) with 95% confidence interval (CI) was used to assess the effect.

**Results:**

The analysis included 7 eligible studies (649 patients). Turmeric and curcumin significantly reduced serum LDL-C (SMD = −0.340, 95% confidence interval [CI]: −0.530 to −0.150, *P* < 0.0001) and TG (SMD = −0.214, 95% CI: −0.369 to −0.059, *P* = 0.007) levels as compared to those in the control group. These may be effective in lowering serum TC levels in patients with metabolic syndrome (MetS, SMD = −0.934, 95% CI: −1.289 to −0.579, *P* < 0.0001), and turmeric extract could possibly have a greater effect on reducing serum TC levels (SMD = −0.584, 95% CI: −0.980 to −0.188, *P* = 0.004); however, the efficacy is yet to be confirmed. Serum HDL-C levels were not obviously improved. Turmeric and curcumin appeared safe, and no serious adverse events were reported in any of the included studies.

**Conclusions:**

Turmeric and curcumin may protect patients at risk of CVD through improving serum lipid levels. Curcumin may be used as a well-tolerated dietary adjunct to conventional drugs. Further research is required to resolve uncertainties related to dosage form, dose and medication frequency of curcumin.

**Electronic supplementary material:**

The online version of this article (10.1186/s12937-017-0293-y) contains supplementary material, which is available to authorized users.

## Introduction

Cardiovascular disease (CVD) is currently the leading cause of mortality worldwide [1]. Major cardiovascular risk factors include age, sex, hypertension, dyslipidemia, obesity, type 2 diabetes mellitus (T2DM), and metabolic syndrome (MetS). Dyslipidemia, hypertension, and insulin resistance, manifesting as T2DM and MetS, promote endothelial dysfunction and vascular inflammation leading to atherosclerosis—the main cause of CVD [2]. An epidemiological study showed that low circulating concentrations of low-density lipoprotein cholesterol (LDL-C) and triglycerides (TG) were associated with a low risk of CVD [3]. Therefore, treatment of dyslipidemia is critical for the prevention of CVD. Statins and fibrates are common lipid-modulating agents and a newer lipid-lowering agent, alirocumab (Praluent), has recently been approved for the treatment of dyslipidemia [2]. Statins are widely used despite their potential to cause serious adverse effects such as myopathies and hepatotoxicity [4, 5]. The estimated incidence of asymptomatic elevation in aminotransferases in patients on statin therapy is less than 2% [6] and the risk of developing rhabdomyolysis (a form of myopathy) is 3 per 100,0000 person-years (associated fatality rate: 10%) [7]. Due to these concerns, it is crucial to focus efforts toward developing more effective drugs and to discover natural agents as alternatives to currently available treatments.

Turmeric (*Curcuma longa*), an Indian spice, is a yellow pigment that is used worldwide in cooking, cosmetics, dyes, and medicines [8]. It is worth noting that turmeric is a frequently used food additive in Southeast Asia, which improves color and flavor of food preparations. Curcumin (chemical name: diferuloylmethane) is an active component of turmeric [9], which has the capacity to interact with hundreds of molecular targets. Several studies have demonstrated the protective effects of curcumin against many chronic diseases, including various cancers, pulmonary disorders, and autoimmune diseases [10]. It has been shown to attenuate oxidative stress [11] and to exert a cardioprotective effect owing to its lipid-lowering properties [12–14]. However, some contradictory results have also been reported [15–21]. In a meta-analysis of 5 randomized controlled trials, overall effects in the entire study population as well as those in a subgroup analysis of subjects with cardiovascular risk factors failed to demonstrate a significant lipid-lowering effect of curcumin [22]. However, the studies included in this meta-analysis had limited sample sizes. Further, most of the randomized controlled trials that have reported positive effects of curcumin on blood lipid levels were published subsequent to the above-mentioned meta-analysis [23–28]. Nonetheless, conflicting reports exist in that some studies have reported promising effects [23–28], whereas others failed to demonstrate any significant effect [15]. Thus, we conducted a meta-analysis of published clinical trials to assess the efficacy and safety of turmeric and cucumin in lowering lipid levels in patients with risk factors for CVD.

## Methods

### Literature search

An online search was carried out for clinical studies published in English language in the following electronic databases: PubMed, Embase, Ovid, Medline and Cochrane Library. All studies published as of November 2016 were eligible for inclusion. Medical Subject Heading (MeSH) terms were used for PubMed, and comparable terms were used for other databases. The search terms were as follows: (curcuminoid OR curcumin OR curcuma OR turmeric OR curcuminoids) AND (hyperlipidemia OR hyperlipidemic OR hypolipidemic OR dyslipidemia OR dyslipidemic OR hypercholesterolemia OR hypercholesterolemic OR hypocholesterolemic OR “low-density lipoprotein” OR “high-density lipoprotein” OR cholesterol OR triglycerides OR hypertriglyceridemia OR hypotriglyceridemic). In addition, we used various synonyms.

### Inclusion and exclusion criteria

Inclusion criteria for research articles included: (1) drug or placebo-controlled parallel randomized trials; (2) subjects with risk factors for CVD, for example, dyslipidemia, T2DM, MetS, hypertension, prediabetes, prehypertension, or obesity; (3) studies that used purified curcumin or a curcuminoid mixture, extracts with determined content of curcumin or curcuminoids, or turmeric powder, regardless of dosage and frequency (this inclusion criteria was established according to the meta-analysis [22] mentioned earlier and a review about the efficacy of turmeric/curcumin for alleviating the symptoms of arthritis [29]); (4) study duration ≥4 weeks; and (5) studies that reported mean ± standard deviation (SD), mean ± standard error (SE) or median (range) of between-group differences between the experimental and control groups at baseline as well as at the end of the trial. Studies were excluded if they featured: (1) a crossover randomized trial design; (2) noncomparative data; (3) lack of outcome measures; and (4) duration of treatment < 4 weeks.

### Efficacy measures

Primary outcome measures included serum levels of LDL-C, high-density lipoprotein cholesterol (HDL-C), TG, and total cholesterol (TC). Treatment safety, measured by adverse effects due to turmeric and curcumin, was defined as a secondary outcome.

### Data extraction

Two independent investigators (SQ and LFH) screened the titles and abstracts of articles initially retrieved on online search of databases, and extracted essential data from eligible full-text articles. Data on study design, patient characteristics, number of patients, treatment regimen, duration of treatment, year of publication, and daily dose of curcumin or turmeric powder were extracted, as were the mean ± SD (or mean ± SE) of all efficacy measures specified earlier. To control relative heterogeneity, only data of efficacy measures pertaining to the study period between 4 weeks and 3 months were extracted. For studies with missing data, authors were sent emails requesting details of these data.

### Study quality

Risk of bias was assessed independently by 2 reviewers (SQ and LFH) using the Cochrane Handbook for Systematic Reviews of Interventions [30]. This tool allows for assessment of the study quality with respect to 6 domains: random sequence generation, allocation concealment, blinding, incomplete outcome data, selective reporting, and other bias. For each domain, the risk of bias was marked as low, unclear, or high.

### Statistical analysis

The meta-analysis was conducted by using Stata version 12.0 (Stata Corporation, College Station, TX, USA). In this analysis, only continuous variables were extracted. If mean values and SD were unavailable, these were calculated from mean values and SE by using the following formulas: mean = mean _post-treatment_ - mean _pre-treatment_; SD = SE × square root n (n: number of participants) and SD = square root [(SD _pre-treatment_)^2^ + (SD _post-treatment_)^2^ - (2*R* × SD _pre-treatment_ × SD _post-treatment)_], assuming a correlation coefficient (*R*) = 0.5 [31]. With use of relevant formulae [32], these values were also calculated from medians and ranges. Because, one of the included studies [23] did not report the unit of outcomes, standardized mean difference (SMD) with 95% confidence interval (CI) was used to present the results of the meta-analysis. Statistical heterogeneity between trials was detected by the Chi-squared and *I*-square (*I* ^*2*^) tests. In the event of significant heterogeneity (*P* < 0.1 and *I* ^*2*^ > 50%), the random-effect model was used for analysis; in other cases, a fixed-effect model was used. Whole effects with *P*-value <0.05 indicated statistical significance between experimental and control groups. SMD value approaching 0 (*P* > 0.05) was judged to have no statistical significance. In contrast, SMD value deviating from 0 (*P* < 0.05) was regarded as a “significant finding”. Subgroup analyses were conducted to explore heterogeneity among studies with respect to underlying disease and form of intervention (turmeric or curcumin) used. As only a single study had reported efficacy measures disaggregated by gender [27], subgroup analyses by gender could not be undertaken. To assess publication bias, funnel plots and Begg’s test were conducted initially. However, as fewer than 10 studies were selected in each meta-analysis, the funnel plots and Begg’s test could not be conducted.

## Results

### Summary of included studies

**Fig. 1:**
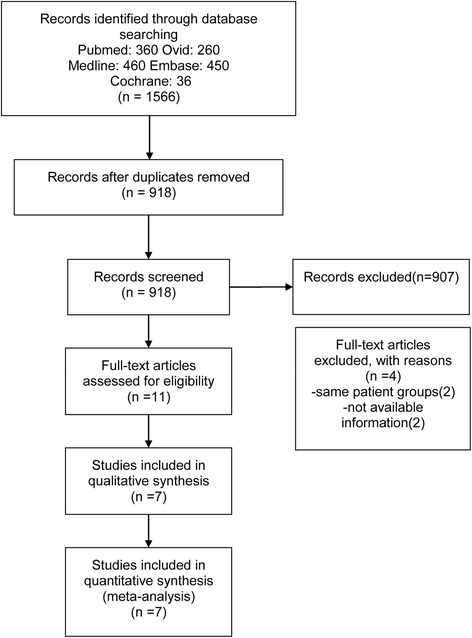
Flow diagram of the literature selection process

Of the 1566 articles retrieved on initial literature search, a detailed evaluation of 11 full-text articles resulted in the elimination of 2 studies [33, 34], due to the use of the same patient groups, and another 2 studies [35, 36], owing to incomplete data (Fig. 1). Consequently, 7 randomized controlled trials (RCTs), including a total of 649 subjects, met the inclusion criteria and were selected for a qualitative analysis.

Basic characteristics of the included RCTs are summarized in Table 1, and the pre- and post-intervention serum lipid parameters are presented in Additional file 1: Table S1. Of the 7 eligible studies, only studies that enrolled patients with T2DM [15, 24, 25, 28] or MetS [23, 26, 27] were included. All studies were conducted in a double-blind manner with the exception of the trials by Selvi et al. [25] and Usharani et al. [15]. Two of the studies were performed in Iran [23, 24] and 2 [15, 25] were conducted in India. The remaining studies were conducted in Pakistan [26], Taiwan [27], and Thailand [28], respectively. The duration of these studies ranged from 4 weeks to 6 months. Specified outcomes were reported from all studies, with the exception of the study by Chuengsamarn et al. [28], for which only serum TG levels were reported. The form of intervention and serum lipid parameters of studies at baseline and after intervention differed.

**Table 1:** Characteristics of studies included in the meta-analysis

Study	Region	Type	Duration	Inclusion criteria		Intervention	*N*	Age	Male
Metabolic syndrome									
Rahmani et al. 2016 [23]	Iran	RCT double-blind	8 weeks	Patients with symptoms of MetS (NCEP-ATP III) and diagnosis of NAFLD	Case	Amorphous dispersion Curcumin (amorphous dispersion preparation, equivalent to 70 mg/d curcuminoids)	37	46.37 ± 11.57	19
					Control	Placebo	40	48.95 ± 9.78	19
Amin et al. 2015 [26]	Pakistan	RCT double-blind	8 weeks	Patients with ≥ 3 features of MetS, prediabetes, dyslipidemia and prehypertension	Case	Turmeric powder 2.4 g/d	63	42.40 ± 13.70	NA
					Control	Placebo	63	41.57 ± 12.80	NA
Yang et al. 2014 [27]	Taiwan	RCT double-blind	12 weeks	Patients with diagnosis of MetS (NCEP-ATP III)	Case	Turmeric extract (equivalent to 1890 mg/d curcuminods)	30	59.03 ± 10.10	12
					Control	Placebo	29	59.61 ± 14.09	17
Type 2 diabetes mellitus									
Rahimi et al. 2016 [24]	Iran	RCT double-blind	3 months	Type 2 Diabetic patients	Case	Curcumin (nano-micelle 80 mg/day)	35	56.34 ± 11.17	17
					Control	placebo	35	60.95 ± 10.77	14
Selvi et al. 2015 [25]	India	RCT	4 weeks	Type 2 diabetic patients	Case	Turmeric powder 2 g/day +Metformin	30	47.00 ± 7.17	30
					Control	Metformin (1 g/day)	30	46.80 ± 6.10	30
Chuengsamarn et al. 2014 [28]	Thailand	RCT double-blind	6 months	Type 2 diabetic patients	Case	Turmeric extract (equivalent to 1500 mg/d curcuminods)	107	59.16 ± 11.04	50
					Control	Placebo	106	59.58 ± 10.71	47
Usharani et al. 2008 [15]	India	RCT	8 weeks	Type 2 diabetic patients	Case	Turmeric extract (equivalent to 600 mg/day curcuminoids)	23	55.52 ± 10.76	12
					Control	Placebo	21	49.75 ± 8.18	11

*MetS* metabolic syndrome, *NAFLD* nonalcoholic fatty liver disease, *NCEP-ATP III* National Cholesterol Education Program Adult Treatment Panel III, a diagnostic guideline of MetS, *RCT* randomized controlled trial, *NA* not available

Values are expressed as mean ± SD

### Data quality

The risk of bias in the individual studies is shown in Additional file 2: Table S2. Overall, these selected studies varied in terms of quality: of the 7 RCTs, 4 were classified as high quality [24, 26–28] and 3 were judged to be of moderate quality [15, 23, 25]. Four studies used appropriate randomization methods, such as a random number table [25, 27] or a computer-generated list of random numbers [24, 28]. Allocation concealment was only used in 4 studies [23, 25, 26, 28]. Five trials used double-blinding of patients and practitioners [23, 24, 26–28]. All studies reported dropout rates and specific reasons for dropout with the exception of the trial by Chuengsamarn et al. [28].

### Meta-analysis

**Fig. 2:**
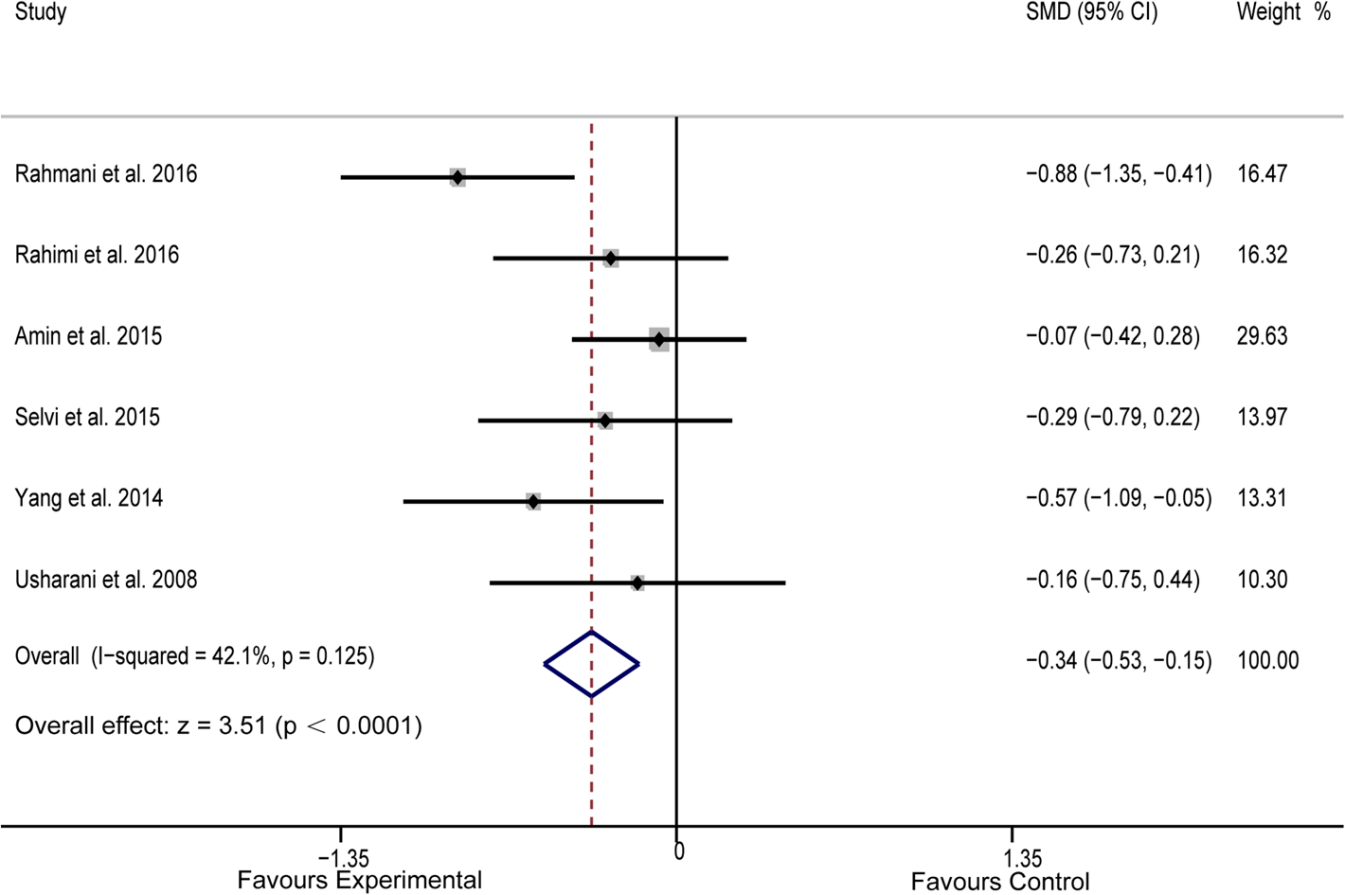
Forest plot of the meta-analysis for comparison of plasma LDL-C concentrations between experimental and control groups

**Fig. 3:**
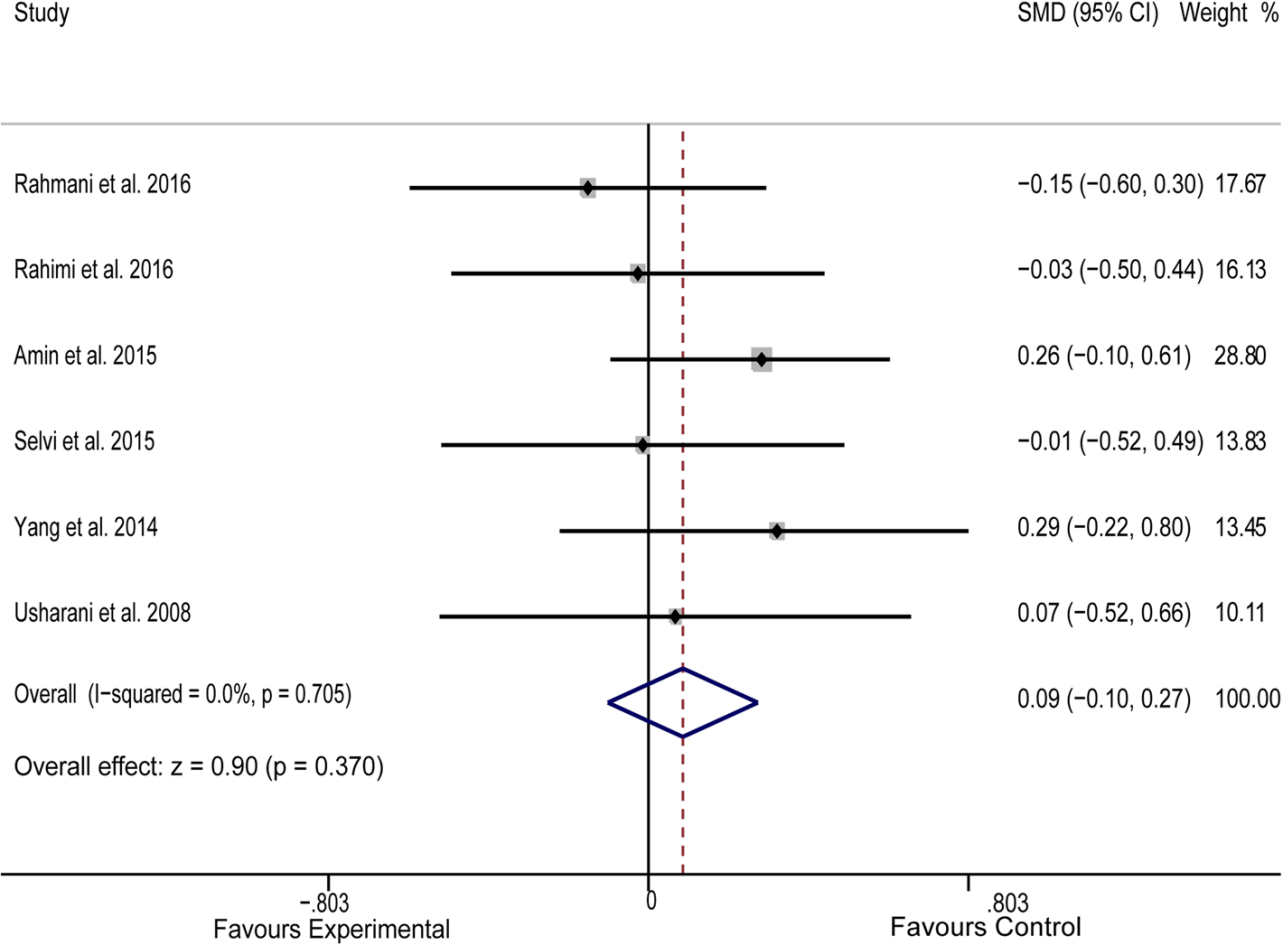
Forest plot of the meta-analysis for comparison of plasma HDL-C concentrations between experimental and control groups

**Fig. 4:**
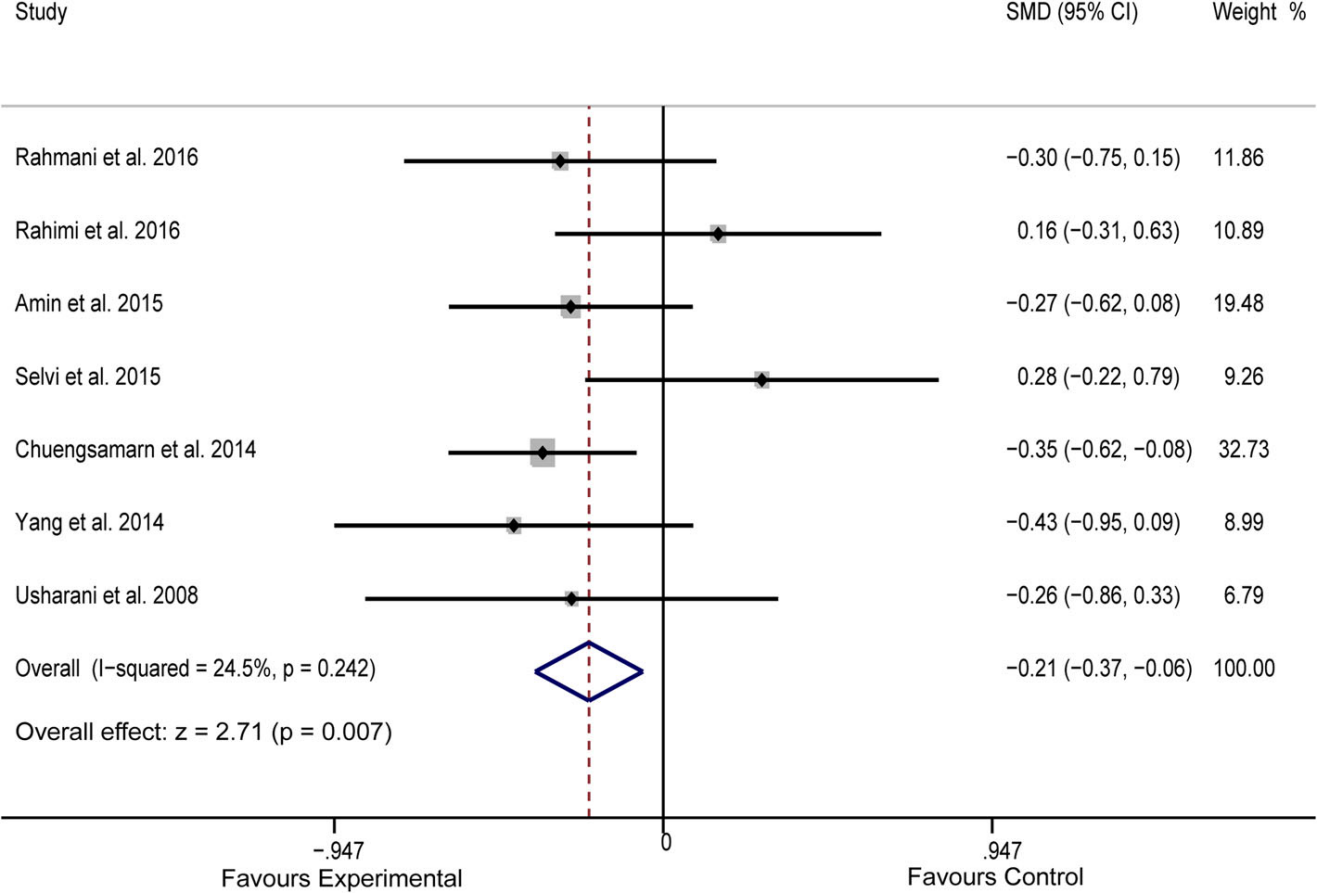
Forest plot of the meta-analysis for comparison of plasma TG concentrations between experimental and control groups

**Fig. 5:**
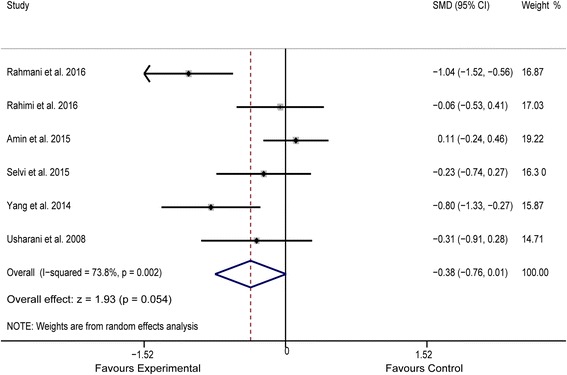
Forest plot of the meta-analysis for comparison of plasma TC concentrations between experimental and control groups

Pooled data from 6 trials [15, 23–27] (*n* = 218 both cases and controls) showed significant efficacy of the study drug in reducing serum LDL-C levels; no significant heterogeneity was observed between these six trials (*P* < 0.0001, *I* ^*2*^ = 42.10%, Fig. 2). Similarly, turmeric and curcumin therapy did not exhibit a favorable effect on serum HDL-C levels (*P* = 0.370, *I* ^*2*^ = 0.00%, Fig. 3). Meta-analysis of data from 7 studies [15, 23–28] indicated an obvious benefit of experimental treatment (*n* = 325) in reducing serum TG levels, compared to that with control treatment (*n* = 324) (SMD = −0.214, 95% CI: −0.369 to −0.059, *P* = 0.007, *I* ^*2*^ = 24.5%, Fig. 4). Although a random-effect model was used, pooled analysis of data from 6 studies [15, 23–27] showed no significant between-group differences in terms of plasma TC concentrations (*P* = 0.054), ostensibly owing to the significant heterogeneity among these studies (*I* ^*2*^ = 73.8%, *n* = 218 for both experiment and control groups, Fig. 5).

### Subgroup analyses

To assess sources of potential bias, we conducted subgroup analyses by underlying diseases and forms of intervention—namely, studies in patients with hyperglycemia (pre-diabetes and T2DM) and MetS (Table 2). Two studies [23, 27] which included patients with MetS revealed significant differences (*P* < 0.0001, *I* ^*2*^ = 0.00%) with respect to serum TC levels between the experimental (*n* = 67) and placebo (*n* = 69) groups. However, pooled data from 4 trials [15, 24–26] showed no significant differences in this respect (*P* = 0.612, *I* ^*2*^ = 0.00%) among patients with hyperglycemia, between the experimental (*n* = 151) and placebo (*n* = 149) groups. With regard to form of intervention, analysis of pooled data from 2 studies [25, 26] comprising 186 subjects showed no favorable effect of turmeric powder therapy on serum TC levels [−0.000 (−0.288 to 0.288), *P* = 0.999, *I* ^*2*^ = 17.1%]. However, 2 studies [15, 27] showed significant benefits of turmeric extract (*P* = 0.004, *I* ^*2*^ = 31.1%) in the experimental group (*n* = 53) as compared to that in the control group (*n* = 50).

**Table 2:** Subgroup analysis of serum total cholesterol (TC) levels

Outcome of interest			No. of	No. of	Effects	SMD	95% CI	Heterogeneity		*P*-value
			study	patients	model			*P* value	*I* ^*2*^ (%)	
			arms							
TC			6	436	Random	−0.375	−0.757 to 0.006	0.002	73.80	0.054
	Form of intervention	Turmeric powder	2	186	Fixed	−0.000	−0.288 to 0.288	0.272	17.10	0.999
		Turmeric extract	2	103	Fixed	−0.584	−0.980 to −0.188	0.228	31.10	0.004*
	Type of disease	MetS	2	136	Fixed	−0.934	−1.289 to −0.579	0.510	0.000	<0.0001***
		Hyperglycemia	4	300	Fixed	−0.059	−0.285 to 0.168	0.561	0.000	0.612

*MetS* metabolic syndrome

*p < 0.05

**p < 0.001

***p < 0.0001

### Side effects of turmeric and curcumin

Five RCTs reported adverse effects in both the control and experimental groups. Rahmani et al. [23] reported that 2 patients experienced simultaneous abdominal pain and nausea, whereas another patient suffered from abdominal pain. Amin et al. [26] reported adverse events such as nausea and dyspepsia, but failed to report the exact number of subjects who experienced these events. Chuengsamarn et al. [28] reported side effects of curcumin in 4 patients: constipation in 2, hot flashes in 1, and nausea in 1 patient. Moreover, 4 patients experienced side effects in the placebo group: vertigo and itching, constipation, and hot flashes in 1 patient each. Selvi et al. [25] reported mild diarrhea in 2 subjects. None of the remaining studies reported any adverse reactions of turmeric and curcumin therapy. No serious adverse reaction induced by turmeric and curcumin was reported in any of the studies included in this meta-analysis.

## Discussion

The epidemic of obesity has contributed to a growing burden of CVD risk factors such as T2DM and MetS [37] (defined as the presence of at least 3 out of the 4 criteria: central obesity, increased blood pressure, high blood sugar levels, and dyslipidemia [38]). Dyslipidemia is a well-established modifiable cardiovascular risk factor. All of the currently available antilipemic therapies have their own inherent shortcomings and disadvantages. Therefore, natural treatments have been investigated as potential therapies for lowering blood lipid levels.

This systematic review of 7 randomized trials of turmeric and curcumin in patients at risk of CVD identified evidence of their beneficial effects on serum TG and LDL-C levels, although no significant difference was found with respect to serum HDL levels. Despite the use of random-effects model to compensate for heterogeneity, no statistically significant benefit was observed with regard to TC (*P* = 0.054). When the analysis was restricted to more homogenous studies based on underlying disease in subjects (hyperglycemia and MetS), a beneficial effect of turmeric and curcumin on serum TC levels was observed in subjects with MetS; however, in subjects with hyperglycemia, this beneficial effect on serum TC levels was not observed. It seems that the natural form (turmeric) and curcumin have more positive effects on patients suffering from MetS. With regard to the forms of intervention, turmeric extract may have a greater beneficial effect on serum TC levels, as compared to that of turmeric in its natural form. However, owing to the limited number of studies, definitive conclusions may not be drawn in this respect. Furthermore, larger scale trials are required among patients with MetS to explore the effect of turmeric extract, even in novel forms, in lowering plasma TC concentrations.

Sahebkar conducted a meta-analysis of 5 RCTs to assess the effects of curcumin on blood lipid levels and found no significant improvements in the lipid profile in any aspect [22]. Several explanations could be tendered to explain why the results of their study were contrary to those of the present study. Firstly, both parallel and crossover randomized trials were selected, and these may have adversely influenced the final results.

Secondly, most of the selected studies were conducted with unformulated curcumin, which is considered to have low bioavailability. Curcumin has poor bioavailability owing to its poor absorption, fast metabolism, and rapid elimination from the body. Some attempts have been made to overcome these deficits, including the use of a piperine (black pepper) adjuvant, liposomal curcumin, nanoparticles, phospholipid complexes, and an amorphous form [39, 40]. Therefore, a hypothesis could be proposed that these novel dosage forms of curcumin may achieve greater clinical effects. To verify this theory, forest plots were conducted initially. Nevertheless, in our present review, only two trials [23, 24] used novel forms, and the preparations used in these studies were dissimilar (amorphous forms were used in one study [23] and nanoparticles were used in the other [24]). Therefore, further research on newer dosage forms of curcumin is required to confirm the hypothesis.

Finally, differences with respect to underlying diseases in the study population may also explain this discrepancy. The authors included healthy participants and patients with various chronic diseases (i.e., coronary artery disease, diabetes, Alzheimer’s disease, or obesity), which may have resulted in different outcomes. Even on performing a subgroup analysis in patients with high cardiovascular risk (acute coronary syndrome, T2DM, or concomitant dyslipidemia and obesity), no significant difference could be identified because coronary artery disease is an end event rather than a risk factor for CVD.

The article by Mohammadi et al. [19] was not included in the meta-analyses because its design as a randomized crossover trial did not fulfill the inclusion criteria. However, the study investigated the hypothesis that curcuminoids (1 g/day for 30 days) lead to a significant reduction in serum triglyceride concentrations in obese individuals. Unlike this study, in a trial among patients with coronary artery disease, although curcumin supplementation decreased serum levels of TC, LDL-C, and TG, there was no obvious difference when compared to placebo [41], possibly due to the small size of the study. Subsequently, a study by Soare et al. found that 900 mg of curcumin did not influence plasma lipid levels in non-obese relatively healthy individuals [42]. Therefore, we tentatively propose that the antilipemic effect of curcumin is evident only in patients who are at a higher risk of cardiovascular morbidity, such as those with MetS, T2DM, and obesity.

Some molecular mechanisms could potentially explain these results. Insulin resistance (IR) is the basic underlying pathology in both T2DM and MetS. Neerati et al. reported that curcumin could counter IR [43]. Through amelioration of metabolic derangement and potential binding of curcumin with peroxisome proliferator-activated receptor gamma (PPAR-γ) as agonist, curcumin could play a preventive role in diet-induced insulin resistance [44]. Moreover, curcumin was shown to increase activation of PPAR-γ [45], which suppressed expression of the LDL-C receptor gene, and could thereby reduce plasma LDL-C concentrations [46]. Because it interacts with multiple targets, including peroxisome proliferator-activated receptor alpha (PPAR-α), PPAR-γ, cholesteryl ester transfer protein (CETP), and lipoprotein lipase, curcumin could probably play a role in reduction of triglyceride levels [47–49]. Furthermore, curcumin is expected to affect both synthesis and catabolism of triglyceride-rich lipoproteins [47–49]. Thus, curcumin supplementation may lower plasma triglycerides and cholesterol concentrations by mitigating the expressions of lipogenic genes [48–50]. Additionally, the lipid-lowering effect of turmeric and curcumin is related to statins. Panahi et al. found that curcumin affected all pathways of cholesterol metabolism that are affected by statin therapy; it also reduced the effective doses of statins, which helped reduce the incidence of serious adverse reactions [51]. Furthermore, curcumin might serve as a valuable adjunct to statin therapy in patients with disordered lipid metabolism [51].

In our meta-analysis, we found that consumption of turmeric and curcumin was safe and well-tolerated in general. This finding is consistent with those of previous studies in human subjects [10], and dosages as high as 8000 mg/day have been shown to be well-tolerated with no apparent toxicity [52].

Several potential limitations of this review need mention. First, the most important limitation may pertain to the interpretability of outcomes. Second, this review did not include unpublished studies or studies published in the “grey literature”. Third, all subjects in the included studies were Asians. Lastly, some data were obtained indirectly, and those could have affected the accuracy of both the overall effects and the results of subgroup analyses.

## Conclusions

Subjects who received turmeric and curcumin experienced a natural cardioprotective effect, with lowering of serum LDL-C and TG levels, as compared to subjects who did not. The efficacy of turmeric and curcumin on serum TC levels remains inconclusive, despite their superior efficacy observed in patients with MetS. A greater effect of turmeric extract in reducing serum TC levels may be observed in patients who are at risk of CVD; however, this finding needs to be confirmed in future studies. No significant change in serum HDL levels was observed.

Because curcumin’s poor bioavailability limits its absorption from dietary sources, novel formulations with enhanced bioavailability are probably required to control dyslipidemia more effectively.

Due to uncertainties related to dosage form, dose and medication frequency, it is premature to recommend the use of turmeric or curcumin in clinical settings. Nonetheless, the analysis does provide a synthesis of the currently available evidence and supports larger scale clinical trials of curcumin.

## Additional files

Additional file 1: Table S1. Serum lipid parameters in studies from before and after intervention. (DOC 64 kb)

Additional file 2: Table S2. Quality of studies assessed by the Cochrane guidelines. (DOC 37 kb)

## Abbreviations

CETP: Cholesteryl ester transfer protein
CI: Confidence interval
CVD: Cardiovascular disease
HDL-C: High-density lipoprotein cholesterol
IR: Insulin resistance
LDL-C: Low-density lipoprotein cholesterol
MeSH: Medical Subject Heading
MetS: Metabolic syndrome
PPAR-α: Peroxisome proliferator-activated receptor alpha
PPAR-γ: Peroxisome proliferator-activated receptor gamma
RCTs: Randomized controlled trials
SD: Standard deviation
SE: Standard error
SMD: Standardized mean difference
T2DM: Type 2 diabetes mellitus
TC: Total cholesterol
TG: Triglycerides
