# Supplementary material for: Efficacy and safety of turmeric and curcumin in lowering blood lipid levels in patients with cardiovascular risk factors: a meta-analysis of randomized controlled trials
Source: Nutr J. 2017 Oct 11;16:68. doi: 10.1186/s12937-017-0293-y (PMC5637251; doi:10.1186/s12937-017-0293-y)
Supplement: Supplementary file 1 — Serum lipid parameters in studies from before and after intervention. (DOC 64 kb) [file 12937_2017_293_MOESM1_ESM.doc]

**Table S1** Serum lipid parameters in studies from before and after intervention

| Study | Outcome | Unit | Experimental | | Control | |
| --- | --- | --- | --- | --- | --- | --- |
|  |  |  | Before | After | Before | After |
| Rahmani et al. | LDL-C | NR | 107.06 ± 31.36 | 95.59 ± 28.22 | 115.57 ± 22.30 | 125.00 ± 24.23 |
|  | HDL-C | NR | 44.26 ± 11.83 | 46.68 ± 10.98 | 42.62 ± 6.67 | 46.72 ± 15.38 |
|  | TC | NR | 198.59 ± 41.76 | 174.38 ± 39.56 | 187.78 ± 32.95 | 196.82 ± 37.04 |
|  | TG | NR | 199.68 ± 91.46 | 173.43 ± 95.44 | 160.20 ± 61.94 | 153.58 ± 50.12 |
| Amin et al. | LDL-C | mg/dl | 111.10 ± 22.20 | 105.40 ± 20.50 | 119.50 ± 27.30 | 138.90 ± 13.20 |
|  | HDL-C | mg/dl | 33.90 ± 8.20 | 35.50 ± 7.00 | 33.70 ± 7.40 | 33.40 ±7.70 |
|  | TC | mg/dl | 176.6 0± 29.60 | 165.00 ± 26.40 | 180.80 ± 23.30 | 179.20 ± 28.90 |
|  | TG | mg/dl | 165.20 ± 40.10 | 153.90 ± 31.40 | 163.60 ± 42.70 | 162.30 ± 32.20 |
| Yang et al. | LDL-C | mg/dl | 120.55 ± 36.81 | 106.51 ± 25.02 | 107.32 ± 24.08 | NA |
|  | HDL-C | mg/dl | 40.96 ± 8.59 | 43.76 ± 9.54 | 41.84 ± 11.80 | NA |
|  | TC | mg/dl | 4.87 ± 1.05 | 4.13 ± 0.84 | 4.60 ± 1.26 | NA |
|  | TG | mg/dl | 226.10 ± 64.99 | 160.79 ± 75.46 | 153.42 ± 80.40 | NA |
| Rahimi et al. | LDL-C | mg/dl | 96.57 ± 33.94 | 91.04 ± 28.72 | 99.78 ± 30.33 | 84.00 ± 12.59 |
|  | HDL-C | mg/dl | 54.30 ± 14.02 | 60.95 ± 15.68 | 60.35 ± 15.96 | 55.00 ± 11.09 |
|  | TC | mg/dl | 163.40 ± 33.94 | 158.62 ± 44.06 | 162.40 ± 38.59 | 149.00 ± 24.62 |
|  | TG | mg/dl | 109(94.75) | 131(60.27) | 142(97.50) | 113(58.00) |
| Selvi et al. | LDL-C | mg/dl | 124.40 ± 170 | 113.20 ± 15.30 | 121.70 ± 26.30 | 116.80 ± 27.00 |
|  | HDL-C | mg/dl | 36.02 ± 8.10 | 38.33 ± 5.04 | 34.30 ± 7.10 | 36.70 ± 6.90 |
|  | TC | mg/dl | 184.60 ± 14.60 | 176.60 ± 14.32 | 181.50 ± 22.30 | 178.10 ± 24.80 |
|  | TG | mg/dl | 120.56 ± 37.10 | 125.70 ± 29.21 | 127.30 ± 33.30 | 123.40 ± 23.80 |
| Chuengsamarn et al. | TG at 3month | mg/dl | 158.24 (40–532) | 166.94 (51–626) | 114.27 (37–372) | 167.24 (51–626) |
| Usharani et al. | LDL-C | mg/dl | 120.35 ± 42.13 | 111.34 ± 37.65 | 125.29 ± 34.94 | 122.18 ± 35.56 |
|  | HDL-C | mg/dl | 38.78 ± 7.69 | 39.91 ± 6.08 | 36.38 ± 7.67 | 37.04 ± 5.92 |
|  | TC | mg/dl | 195.00 ± 41.16 | 185.34 ± 34.35 | 196.95 ± 35.72 | 198.76 ± 35.09 |
|  | TG | mg/dl | 176.39 ± 27.61 | 165.26 ± 25.78 | 170.14 ± 47.54 | 168.14 ± 47.10 |

**Data are shown as mean ± SD or median (interquartile range);**

**LDL-C: low-density lipoprotein cholesterol; HDL-C: high-density lipoprotein cholesterol; TC: total cholesterol; TG: triglycerides; NA: not available.**
