# Supplementary material for: Efficacy and safety of turmeric and curcumin in lowering blood lipid levels in patients with cardiovascular risk factors: a meta-analysis of randomized controlled trials
Source: Nutr J. 2017 Oct 11;16:68. doi: 10.1186/s12937-017-0293-y (PMC5637251; doi:10.1186/s12937-017-0293-y)
Supplement: Supplementary file 2 — Quality of studies assessed by the Cochrane guidelines. (DOC 37 kb) [file 12937_2017_293_MOESM2_ESM.doc]

**Table S2** Quality of studies assessed by the Cochrane guidelines

| Study | Random sequence | Allocation | Blinding | Incomplete | Selective | Free of other bias |
| --- | --- | --- | --- | --- | --- | --- |
|  | generation | concealment |  | outcome data | reporting |  |
| Rahimi et al. 2016 | L | U | L | L | L | H |
| Rahmani et al. 2016 | U | L | L | L | U | U |
| Selvi et al. 2015 | L | L | H | L | U | U |
| Amin et al. 2015 | U | L | L | L | L | U |
| Yang et al. 2014 | L | U | L | L | L | U |
| Chuengsamarn et al. 2014 | L | L | L | U | L | U |
| Usharani et al. 2008 | U | U | H | L | L | U |

**H: high risk of bias; L: low risk of bias; U: unclear or unrevealed risk of bias.**
